# The Impact of Statin Therapy on Cardiovascular Outcomes in Patients With Diabetes: A Systematic Review

**DOI:** 10.7759/cureus.47294

**Published:** 2023-10-18

**Authors:** Nia Uswanti Binti Usman, Tanusha Winson, Prithvi Basu Roy, Vitrag N Tejani, Sukhmeet S Dhillon, Nanush Damarlapally, Binay K Panjiyar

**Affiliations:** 1 Internal Medicine, University of Brawijaya, Malang, IDN; 2 Internal Medicine, Asian Institute of Medicine, Science and Technology (AIMST) University, Sungai Petani, MYS; 3 Cardiology, Kali Pradip Chaudhuri (KPC) Medical College and Hospital, Kolkata, IND; 4 Pharmacology, Dr. N. D. Desai Faculty of Medical Science and Research, Dharmsinh Desai University, Nadiad, IND; 5 Internal Medicine, Parul Institute of Medical Sciences and Research, Parul Sevashram Hospital, Parul University, Vadodara, IND; 6 Internal Medicine, Baba Farid University of Health Sciences, Patiala, IND; 7 Health Sciences, Coleman College for Health Sciences, Houston, USA; 8 Internal Medicine, Harvard Medical School, Boston, USA; 9 Internal Medicine, California Institute of Behavioral Neurosciences and Psychology, Fairfield, USA

**Keywords:** cardiovascular on diabetes patients, statin on cardiovascular, diabetes patients, cardiovascular outcome, statin therapy

## Abstract

Cardiovascular disease (CVD) is the primary cause of death all over the world, especially due to myocardial ischemia caused by atherosclerosis that blocks cardiac arteries and leads to arrhythmia and other cardiac diseases. Meanwhile, diabetes mellitus (DM) and elevated cholesterol level are the risk factors for cardiovascular (CV) disease. This noncommunicable disease has become a main concern for us as cardiovascular disease develops in a slow manner without any symptoms in the early stage. Early prevention and intervention have a major impact on improving the outcome of cardiovascular health in diabetic patients. Controlling cholesterol level by administering statin has shown some beneficial impacts in reducing the risk of cardiovascular disease in patients with DM. This study used a systematic literature review (SLR) approach to give an overview of the current literature and to analyze the effects of statin therapy on cardiovascular outcomes in patients with DM. The literature search was conducted in PubMed and Google Scholar databases. The total number of articles included in the present review is six, obtained from reputable journals published between 2013 and 2023, and we only focused on reviewing six articles for in-depth analysis. The evidence we collected showed a positive outcome in terms of cardiovascular health in persons with DM after statin therapy. However, there are several risk factors that interfere with the effectiveness of statin in diabetic patients.

## Introduction and background

Cardiovascular disease (CVD) and diabetes mellitus (DM) are both related to each other. Most diabetic patients will develop CVD in the future if there is no prevention taken to lower the risk of it happening. CVD is the leading cause of death globally. Worldwide, around 17.9 million people die from this disease [1]. Around 2.6 million deaths are estimated to be caused by elevated cholesterol, and another 29.7 million people experience disability yearly. The prevalence of coronary heart disease is 1.5% and increases with age [2].

In diabetic patients, there is insulin resistance, which causes hyperglycemia that eventually causes metabolic abnormalities such as dyslipidemia, where there is a high cholesterol level, and other causes that favor the formation of atherosclerosis in the coronary artery [3,4]. The prevalence of CVD increases in the presence of DM. Atherosclerosis can cause myocardial ischemia due to partial or completely blocked blood vessels, which circulate and supply blood to the cardiac muscle, and thus disturb the cardiovascular (CV) function, in which blood is pumped all over the body [3,5]. These blocked blood vessels will cause a heart attack with chest pain due to myocardial ischemia.

The complete blockage of the coronary artery will result in ST-segment elevation myocardial infarction (STEMI). Patients with non-ST-segment elevation myocardial infarction (NSTEMI) have a partial blockage with no visible ST elevation on ECG. STEMI patients have a greater mortality risk than NSTEMI patients [6]. It is crucial to curb the number of CVD mortality by controlling cholesterol levels, measured by low-density lipoprotein cholesterol (LDL-C) level of <100 mg; this lipid profile is considered a reversible risk factor. Elevated cholesterol levels can accumulate, narrowing the blood vessels, and plaque them, increasing the risk of CVD. To reduce LDL-C levels, we use statin as a gold standard treatment. Statin is a 3-hydroxy-3-methylglutaryl coenzyme A (HMG-CoA) reductase inhibitor [7]. In the population of people aged more than 40 years old with DM, the usage of statin has been shown to decrease cardiovascular events and likely reduce mortality. Aggressive reduction in low-density lipoprotein cholesterol (LDL-C) reduces cardiovascular event rates, with each 1 mmol/L reduction in LDL-C reducing the annual rate of such major vascular events by approximately a fifth [8]. Although statin use can lower the risk of CVD in patients with DM, cardiovascular morbidity and mortality remain high in most patients with diabetes [9].

Diabetes increases the risk for vascular events regardless of age, and this increase is even more pronounced in people who have had diabetes in the long term or when multiple cardiovascular risk factors coexist, as is common among older people; this vascular dysfunction will promote the process of atherosclerosis in CVD. Other cardiovascular risk factors are hypertension, hypercholesterolemia, tobacco use, and obesity; the earlier onset age of DM and a more extended period of DM diagnosis are also associated with a higher chance of CVD [10,11]. Our objective in this systematic review is to evaluate the available evidence of the effectiveness of statin on cardiovascular outcomes in diabetic patients by answering the following question: can statin help reduce the risk of CVD in patients with DM?

## Review

Methods

**Figure 1:**
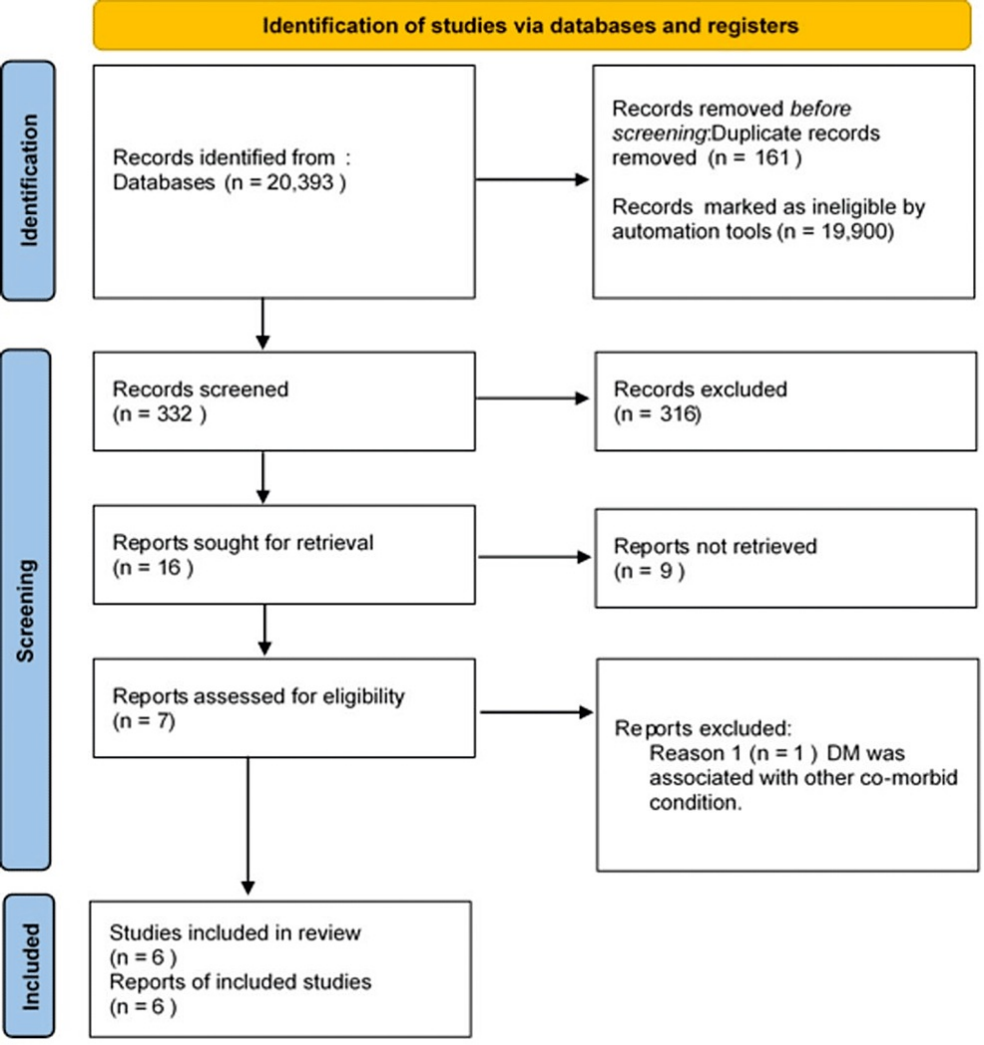
PRISMA flow diagram illustrating the search strategy and study selection process for the systematic review. PRISMA, Preferred Reporting Items for Systematic Reviews and Meta-Analyses; DM, diabetes mellitus

This review highlights clinical studies regarding the prescription of statin on CVD outcomes in patients with DM. We excluded animal studies and publications that only discussed the methodology of statins without presenting clinical data. The review follows the guidelines for Preferred Reporting Items for Systematic Reviews and Meta-Analyses (PRISMA) 2020 (Figure 1) while only taking data collected from published papers, eliminating the need for ethical approval [12].

Systematic literature search and study selection

A thorough search has been conducted to find relevant articles and publications using PubMed, as well as Medical Literature Analysis and Retrieval System Online (MEDLINE) and Google Scholar. We searched for studies mentioned in review papers, editorials, and commentaries on PubMed. Nevertheless, we continued searching for additional studies that fulfilled our inclusion criteria.

We independently reviewed a list of abstracts for inclusion using specific criteria. The criteria included statin therapy, focusing on CVD outcomes of DM patients in the study. We excluded review papers and animal studies.

Inclusion and exclusion criteria

We accepted specific criteria to include and exclude participants to achieve our study goals. Our criteria can be summarized in Table 1.

**Table 1:** The criteria adopted during the literature search process.

Inclusion Criteria	Exclusion Criteria
Human studies	Animal studies
2013-2023	Only methodological studies explaining programming details
English text	Non-English texts
Gender: all	Age: <40 years
Age: >40 years of age	Studies involving clinical data other than cardiovascular health on patients with diabetes mellitus
Free papers	Papers that need to be purchased

Search strategy 

Table 2 shows the search strategy, search engines used, and the number of results displayed.

**Table 2:** The search strategy, search engines used, and the number of results displayed. MeSH, Medical Subject Heading; MEDLINE, Medical Literature Analysis and Retrieval System Online

Database	Search Strategy	Results
PubMed	((Statin therapy[Title/Abstract]) OR (Statin on cardiovascular[MeSH Terms]) OR (Cardiovascular on Diabetes patients[MeSH Terms]) AND ("2013/01/01"[Date - Publication] : "3000"[Date - Publication]))	7,467
PubMed (with filters)	((Statin therapy[Title/Abstract]) OR (Statin on cardiovascular[MeSH Terms]) OR (Cardiovascular on Diabetes patients[MeSH Terms]) AND ("2013/01/01"[Date - Publication] : "3000"[Date - Publication]) AND (y_10[Filter]) AND (ffrft[Filter]) AND (clinical trial[Filter] OR meta-analysis[Filter] OR randomized controlled trial[Filter] OR review[Filter] OR systematic review[Filter])). Filters applied: free full text, clinical trial, meta-analysis, randomized controlled trial, review, systematic review, in the last 10 years, humans, English, female, male, middle aged + aged 45+ years, and MEDLINE	393
Google Scholar	(Statin therapy OR Statin on cardiovascular OR Cardiovascular on Diabetes patients). Filters applied: studies from 2013 to 2023 and review articles	20,000

Quality appraisal 

To ensure the reliability of our chosen papers, we use various quality assessment tools. We used the PRISMA checklist and Cochrane bias tool assessment for randomized clinical trials for systematic reviews and meta-analyses. Non-randomized clinical trials were evaluated using the Newcastle-Ottawa tool scale. We assessed the quality of qualitative studies, as shown in Table 3, using the Critical Appraisal Skills Programme (CASP) checklist.

**Table 3:** Quality appraisal tool used. PRISMA: Preferred Reporting Items for Systematic Reviews and Meta-Analyses

Quality Appraisal Tools Used	Type of Studies
Cochrane bias tool assessment	Randomized controlled trials (RCT)
Newcastle-Ottawa tool	Non-RCT and observational studies
PRISMA checklist	Systematic reviews

Results

Following the search through three selected databases, PubMed, MEDLINE, and Google Scholar, we extracted 20,393 articles. We then carefully analyzed each paper, applied our specific criteria, and chose not to utilize 19,900 due to duplicates or unsatisfactory titles and abstracts. We closely examined the remaining 16 papers and excluded nine more as their content did not meet our inclusion criteria. Finally, we conducted a thorough quality check on the remaining seven papers, which all met our criteria. These six articles are included in our final systematic review. Table 4 provides a detailed description of each.

**Table 4:** Summary of the results of the selected papers. CVD, cardiovascular disease; DM, diabetes mellitus

Author and Year	Country	Study Design	Databases Used	Conclusions
Ikhsan et al., 2022 [13]	Indonesia	Cross-sectional studies	Google Scholar	DM patients face CVD risk influenced by gender, age, smoking, diabetes, blood pressure, and cholesterol level
Bertoluci and Rocha, 2017 [14]	Brazil	Review	Google Scholar	The improved stratification of diabetes patients enhances treatment quality
Johansen et al., 2014 [15]	USA	Database highlights	Google Scholar and PubMed	There are a considerable number of people that can benefit from statin but did not receive the therapy
Nichols et al., 2019 [16]	USA	Observational longitudinal cohort study	Google Scholar	Despite statin use, CVD risk is higher in DM patients due to high triglyceride level
Ramos et al., 2018 [17]	Spain	Retrospective cohort study	Google Scholar	The effect of statin therapy shows the reduction of CVD risk in DM patients younger than 85 years old
Jung, 2021 [18]	Korea	Retrospective cohort study	PubMed	Proper risk assessment and regular statin use in patients at high predicted risk would reduce outcome risks

Discussions

Statin reduces the risk of mortality and CVD in individuals at high cardiovascular risk. Statin users reported filling two or more statin prescriptions from a pharmacy during 2010 [19]. According to Johansen et al., that has created multiple logistic regression models for statin use as the dependent variable, with cardiovascular risk factors as independent variables. However, many people at risk of cardiovascular events, including individuals with diabetes, were not receiving statin as an agent that can reduce CVD risk. The undertreatment is due to a focus on the hyperlipidemia profile and not enough on cardiovascular risk [15]. Guidelines, public health messages, and direct-to-consumer advertising have anchored statin to lower cholesterol levels rather than reduce cardiovascular risk. This overdependence on cholesterol levels shows that those with hyperlipidemia but without DM or heart disease are more likely to be given statin than those without hyperlipidemia who have diabetes or heart disease [20]. Given that individuals with heart disease or diabetes are at considerably higher cardiovascular risk, this suggests that statin use is strongly driven by hyperlipidemia rather than overall cardiovascular risk.

Statin significantly reduced cardiovascular events by about seven per 1,000 people treated for one year. Several studies support the benefit of statin on CVD. The Justification for the Use of Statins in Prevention: An Intervention Trial Evaluating Rosuvastatin (JUPITER) trial found a 39% reduction in CVD in statin-treated patients over 70 years old but no significant improvement in mortality [21]. Two meta-analyses also addressed the statin effect on CVD. Savarese et al. [22] and Teng et al. [23] found that statin significantly reduces myocardial infarction incidence in patients older than age 65 years. Additionally, Ramos et al. discovered that in diabetic patients, statin medication lowers the risk of atherosclerotic cardiovascular disease (CVD), but its effects disappear in nonagenarians after 85 years [17].

In cardiology practices, statins are prescribed for only about 62% of patients aged 40-75 years, of whom just over 50% continue statin use for an extended period [24]. Statins are recommended for primary CVD prevention in diabetes patients aged 40 years and older and for secondary CVD prevention in all adults [25]. Only 40%-60% of diabetic individuals achieve LDL-C values below 100 mg/dL [26,27]. A regular use of statin was defined as using statin for more than two-thirds of each statin therapy period (from the time medication was started to the year of follow-up), intermittent use as between one-third and three-quarters of each period, occasional use as less than one-third of each period, and nonuse as less than 90 days [28]. According to a study by Jung, regular users saw a 43% risk reduction for major CVD events compared to nonusers, which was close to the secondary analysis's 44% risk reduction (i.e., regular versus occasional users) and the 24%-44% CVD risk reductions seen in statin trials for primary prevention [18]. According to this study, a suitable risk assessment and consistent statin therapy in individuals with high anticipated risks might lower outcome risks.

The primary lipid to target in people with diabetes to prevent CVD is LDL-C. Nevertheless, it is common for diabetic people to have high triglyceride (TG) levels [25,29]. The American Diabetes Association (ADA) stated that a post hoc analyses of clinical trials for reducing LDL-C suggest that TG levels are also related to CVD [30]. This statement is supported by Nichols et al. that despite high-density lipoprotein cholesterol (HDL-C) adjustments and statin-controlled LDL-C levels, CV event rates were higher than usual in diabetic patients with elevated TG levels [16]. To account for this finding, mean TG levels were shown to be strongly linked with all-cause mortality in one Italian research of diabetic patients receiving lipid-lowering medication, regardless of other cardiometabolic risk factors [31]. The difference in TG level likely contributed to the extra risk shown in individuals with high TG levels.

Patients with DM have a two to four times higher risk of developing incident coronary heart disease and ischemic stroke and a 1.5-3.6 times higher chance of dying. Diabetes has long been regarded as an "equivalent cardiovascular risk." This claim was previously supported by Finnish research, which found that DM patients without coronary heart disease episodes had similar coronary mortality to non-diabetic individuals with a history of coronary events [32,33]. The length of diabetes is a significant factor in determining the risk of CVD. Patients who have had diabetes for more than 10 years may be at an incredibly high risk. Diabetes age of onset and duration are connected; diabetes diagnosis at an early age may impart an extra risk irrespective of diabetes duration [34]. According to Bertoluci and Rocha, the stratification of diabetic individuals increases the precision in predicting future cardiovascular events, silent ischemia, and subclinical coronary artery disease. To prevent overtreating lower-risk individuals, stratification separates greater-risk patients from lower-risk patients who may require intensive CVD prevention [14].

The adoption of a risk factor-based strategy to determine the start of statin medication is encouraged by the 2016 American Diabetes Association (ADA) Standards of Diabetes Care. In essence, it advises risk stratification using age, the history of cardiovascular events, and the presence or absence of risk factors. A family history of early CVD, LDL-C of >100 mg/dL, high blood pressure, smoking, overweight, and obesity are all ADA risk factors [35].

The American Heart Association classifies the statin intensity as low, moderate, and high intensities as shown in Table 5 [36].

**Table 5:** American College of Cardiology/American Heart Association classification of statin intensity relative to dose prescribed.

Low-Intensity Statin Therapy	Moderate-Intensity Statin Therapy	High-Intensity Statin Therapy
Simvastatin 10 mg	Simvastatin 20-40 mg	Atorvastatin 40-80 mg
Pravastatin 10-20 mg	Pravastatin 40-80 mg	Rosuvastatin 20-40 mg
Lovastatin 10 mg	Atorvastatin 10-20 mg	
Fluvastatin 20-40 mg	Rosuvastatin 5-10 mg	
Pitavastatin 1 mg	Lovastatin 40 mg	
	Fluvastatin 40 mg twice a day	
	Pitavastatin 2-4 mg	

High-intensity statin treatment should be administered to all patients with certain CVD events, regardless of age. High-intensity statin medication is advised for people between the ages of 40 and 75 who have cardiovascular risk factors but have not experienced CVD events. Depending on the inclination and tolerance of the particular patient, either moderate- or high-intensity statin medication might be recommended for older or younger individuals with risk factors. Moderate statin medication is recommended for older adults without risk factors or CVD occurrences. The ADA believes that lifestyle treatment alone is more appropriate in younger individuals without CVD or risk factors. ADA recently recommended for those recently diagnosed with acute coronary syndrome that it is appropriate for these individuals to use high-intensity statin [14,35].

Recent research shows that the risk of CVD in people with DM is highly heterogeneous and not always comparable to those with prior cardiovascular disease. A meta-analysis of 13 epidemiological studies with 45,108 patients with and without DM found that the risk of CVD was 43% lower in DM patients without a history of myocardial infarction than in non-diabetics [37].

Limitations

It should be noted that our literature review has certain limitations. Firstly, we focused solely on English articles that were published within the last decade and were aimed at individuals aged 40 years and above. Secondly, we only utilized free articles for our analysis, and our study was confined to examining the impact of statin on patients with cardiovascular and diabetes conditions. It is clear that further research needs to be conducted to arrive at a definitive conclusion.

## Conclusions

Our study emphasizes the effect of statin medication on cardiovascular results in diabetic individuals. This study on statin therapy aims to maximize the use of statin as a CVD prevention drug.

Diabetes is a problem for CVD risk, even though many factors affect how well a statin works to minimize cardiovascular impact. The analysis of the elements that affect a statin's efficacy has been done from various study publications. Some researchers have effectively demonstrated that statin can reduce the risk of heart disease and diabetes can increase the risk of CVD events if they are not used in conjunction with statin therapy.
